# Baron Philippe Boyer

**Published:** 1860-01

**Authors:** 


BARON PHILIPPE BOYER died recently at Paris, from invagination of the bowel. He was one of the surgeons of the Hôtel-Dieu, an adjunct professor of the faculty of medicine, an officer of the Legion of Honor, and the editor of a new edition of his father's great work, " Traité des Maladies Chirurgicales," the seventh and last volume of which was published in 1853.

